# Diffuse Subcutaneous Emphysema, Pneumomediastinum, and Pneumothorax following Robotic Assisted Laparoscopic Hysterectomy

**DOI:** 10.1155/2017/2674216

**Published:** 2017-10-12

**Authors:** Laryssa Patti, William Haussner, Grant Wei

**Affiliations:** ^1^Rutgers Robert Wood Johnson Medical School, Department of Emergency Medicine, 1 RWJ Place, MEB 104, New Brunswick, NJ 08901, USA; ^2^Rutgers Robert Wood Johnson Medical School, 675 Hoes Lane, Piscataway, NJ 08854, USA

## Abstract

Robotic assisted laparoscopic surgery is becoming more widely available, but despite its multiple benefits, it is not without risk. This case is of a 62-year-old female who presented to the emergency department for dyspnea two days after robotic assisted laparoscopic hysterectomy. Physical exam revealed diffuse facial, neck, upper extremity, torso, and lower extremity crepitus, which was diagnosed as diffuse subcutaneous air on computed tomography (CT). Imaging also revealed right apical pneumothorax and pneumomediastinum. The patient progressively improved over one month, with resolution of symptoms.

## 1. Introduction

Robotic laparoscopic surgery is currently widely performed in both abdominal and gynecologic surgery, as it provides better access and visualization to surgical sites, especially those of the retroperitoneum [1]. Typically, the first step in laparoscopic surgery is to enter the abdomen and insufflate it with gas to allow for visualization of structures and space for surgical manipulation [2]. Traditionally, carbon dioxide (CO_2_) is used for insufflation because it is absorbed faster than air and thus allows for rapid insufflation and desufflation. It is also associated with decreased postoperative abdominal pain and distention as compared to air [3]. A rare but concerning complication of carbon dioxide insufflation is subcutaneous emphysema [4]. Although some studies state that this risk is minimal [3], others have shown that longer intraoperative times and prolonged insufflation of the peritoneum can increase incidence of this complication [4] and that retained carbon dioxide postoperatively can alter acid-base dynamics and cause cardiopulmonary collapse, especially in patients with decreased cardiac, pulmonary, or renal function [2].

## 2. Case

This patient is a 62-year-old female who presented to the emergency department (ED) for chest pain two days following robotic assisted laparoscopic supracervical hysterectomy with sacral colpopexy for a history of uterovaginal prolapse. Per operative report, surgery was prolonged due to incomplete instrument count at the end of the surgery, requiring X-ray to retrieve the missing instrument. On postoperative day one, the patient had an intraoperatively placed Foley catheter removed, passed flatus, and tolerated liquids. She was discharged on that day with oral pain medication and a bowel regimen.

In the emergency department, the patient described chest pain as substernal, pleuritic, and radiating to both shoulders. Chest pain was associated with mild dyspnea that was exacerbated by exertion. She reported a “crunching” sensation in her skin on her torso and neck. Her only past medical history beyond the uterovaginal prolapse was a history of osteoporosis treated with raloxifene.

Her vital signs at triage were oral temperature 36.8 degrees Celsius, pulse 91 beats per minute, respiratory rate 16 breaths per minute, blood pressure 140/67 mmHg, and oxygenation 96% on room air. On exam, the patient had diffuse crepitus, including at the angle of mandible, neck, anterior chest wall, abdomen, and bilateral thighs. Breath sounds were symmetric and nondiminished. Heart sounds were regular and without murmur. Her abdominal exam also revealed well-healing surgical incisions that were clean, dry, and intact.

**Figure 1:**
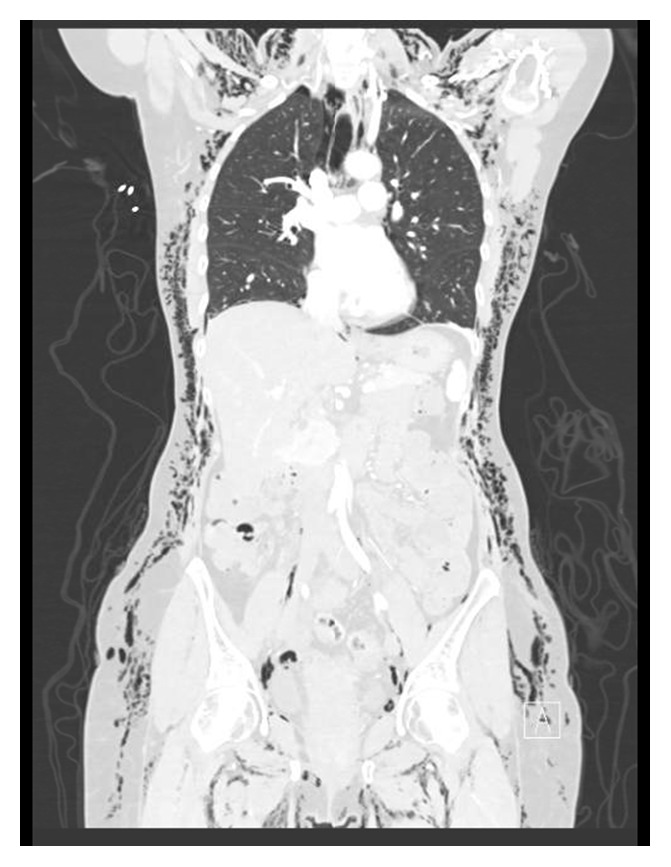
Massive subcutaneous free air.

**Figure 2:**
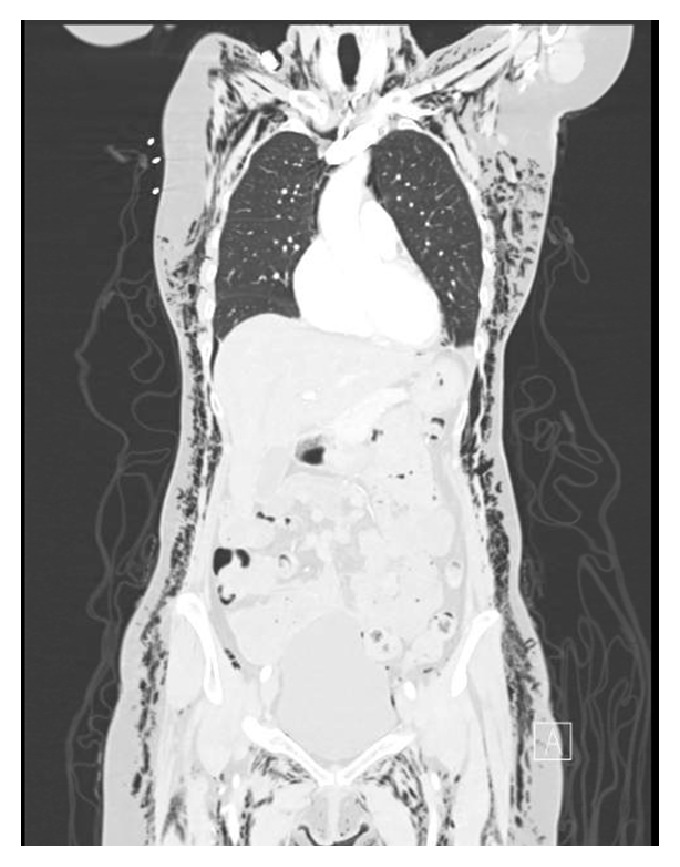
Massive subcutaneous free air, pneumoperitoneum, and pneumomediastinum.

**Figure 3:**
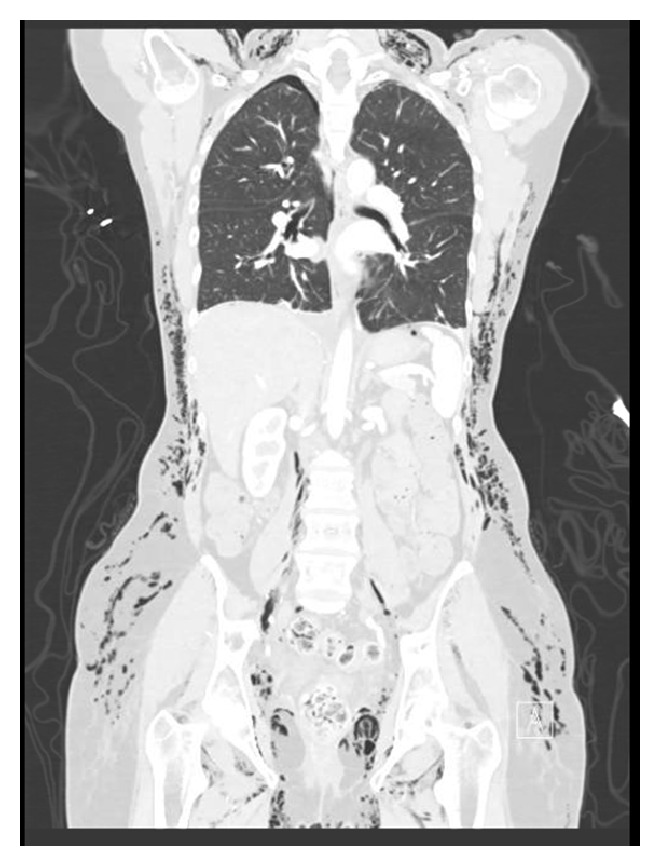
Massive subcutaneous free air, pneumoperitoneum, and small right apical pneumothorax.

Lab results revealed a white blood cell count 7.1 × 10^3^/uL, hemoglobin 11.9 g/dL, and hematocrit 35.4%. These values were similar to those from 2 days earlier. Sodium was 142 mEq/L, potassium 3.8 mEq/L, chloride 103 mEq/L, and bicarbonate 28.5 mEq/L. ProBNP and troponin T were both within normal limits. ECG showed sinus rhythm at 69 beats per minute, with normal intervals, normal axis, and QTc 417 milliseconds. CT angiography of the chest, abdomen, and pelvis with and without contrast showed diffuse, extensive subcutaneous emphysema (Figure 1) involving the entirety of the thoracic and abdominal pelvic subcutaneous tissues and extending posteriorly and laterally, associated with free air dissecting along the retroperitoneal plane. CTA also revealed pneumoperitoneum, pneumomediastinum (Figure 2), and a small right apical pneumothorax (Figure 3).

The patient was treated with oxygen via nasal cannula as treatment for the small pneumothorax and was admitted to the gynecology surgical service for observation. Her serum bicarbonate remained within normal limits (26.2–28.5 mEq/L) and chest X-ray approximately fourteen hours after initial CTA showed no significant change in subcutaneous emphysema and pneumothorax. A venous duplex study was performed for concern for deep venous thrombosis of the lower extremities. It was technically limited due to subcutaneous emphysema in the upper legs, however, showing no thrombosis in the visualized distal deep veins. The patient was discharged on hospital day two without complication.

## 3. Discussion

Many of the risks associated with laparoscopic surgery are rare but result from the creation of pneumoperitoneum by carbon dioxide insufflation. Increased airway pressure, decreased functional residual capacity, and subcutaneous emphysema are usually benign and self-limited. Case reports show most cases resolve within 1 week [5, 6].

However, there is a risk of carbon dioxide absorption into the blood. This resulting hypercarbia is measured by respiratory circuits in the pons and medulla via changes in hydrogen ion concentrations. Specifically, acid sensitive chemoreceptors, present in both the peripheral carotid and aortic bodies and the central nervous system, measure changes in hydrogen ions secondary to increases in CO_2_ tension. These signals are sent to the solitary nucleus of the medulla, which has outputs to the dorsal and ventral respiratory groups of the medulla, and subsequently increase rate and depth of breathing [7].

Additionally, increased levels of CO_2_ can cause sympathetic stimulation. It is hypothesized that the dorsal and ventral respiratory groups of the medulla interact with neurons from the sympathetic nervous system at the level of the pons. This allows for positive regulation of the sympathetic nervous system by the respiratory center of the brainstem during times of increased CO_2_. Consequently, along with an increase in respiratory rate and depth, hypertension and tachycardia can also result in secondary sympathetic stimulation from the respiratory groups [8]. Sympathetic modulation may result in cardiovascular collapse if severe, especially in patients with cardiopulmonary or renal comorbidities [2].

Similarly, pneumothorax and pneumomediastinum are rare complications that occur in less than 0.5% of laparoscopic gynecologic surgery, yet they are potentially life-threatening due to their impairment of adequate oxygenation [9]. It is hypothesized that these may occur in patients with preexisting diaphragmatic defects [10]. However it is possible that the insufflation itself can create a diaphragmatic defect, especially if the surgery is prolonged [11].

Recent literature has suggested the use of helium insufflation instead of CO_2_ in laparoscopic surgery. It was demonstrated that cardiopulmonary derailments similar to those in this case occurred less frequently when helium was used instead of CO_2_, air, or nitric oxide. However, complications from helium insufflation were identical to those of carbon dioxide, so further research is needed to elucidate if a change to helium insufflation is warranted [12].
